# Age- and sex-specific reference intervals for trace elements in infants and children: a multi-center study in Lincang, China

**DOI:** 10.3389/fped.2025.1547429

**Published:** 2025-09-29

**Authors:** Junqiang Li, Zhongguo Chen, Xuezu Zhang, Zhaobing Fu, Min Qin

**Affiliations:** ^1^ Department of Neonatology, Lincang Maternity and Child Health Hospital, Lincang, China; ^2^ Department of Paediatrics, The People’s Hospital of Lincang, Lincang, China; ^3^ Department of Paediatrics, Maternity and Child Health Hospital of Linxiang District, Lincang, China; ^4^ Administrative Department of Lincang Maternity and Child Health Hospital, Lincang, China

**Keywords:** pediatric, reference intervals, trace elements, age partitioning, multi-center study

## Abstract

**Background:**

We used an algorithm to determine age- and sex-specific reference intervals (RIs) for copper, zinc, calcium, magnesium, iron, and lead in blood. Data were collected from three health screening centers.

**Methods:**

The data were obtained from the trace element test results of 3933 outpatients aged <18 years at three health examination centers in Lincang City between June 2014 and May 2024. Atomic absorption spectrometry was employed to measure the trace element. Participants were divided into subgroups at 1-year intervals according to age and gender. Decision trees were constructed by the Classification and Regression Tree method to determine the optimal age segmentation point. Harris-Boyd and Lahti methods were used to assess the appropriateness of age segmentation.

**Results:**

After the appropriate segmentation points are determined, the refineR algorithm is applied to calculate RIs. After data cleaning, 3933 samples were analyzed for age subgroup partitioning of trace elements from 1 month through 18 years. The difference between the age sub-groups was statistically significant according to the Harris-Boyd method and the Lahti method. Blood copper and calcium levels gradually decreased with months and blood zinc and iron concentration gradually increased with months. After gradually decreasing with months, blood pb levels in boys and girls tended to peak at 15 years and beyond. Blood magnesium levels remained stable.

**Conclusions:**

We have established RIs for six trace elements for children, and the methods we use provide reference for laboratories around the world.

## 1 Introduction

Essential trace elements such as calcium (Ca), iron (Fe), copper (Cu), magnesium (Mg), and zinc (Zn) are involved as metal cofactors in the function of many enzymes and proteins, and they are essential in maintaining the metabolism (1, 2). As key components of metalloenzymes, essential elements are involved in vital biological functions, such as oxygen transport, free radical scavenging, and hormonal activity (3). The animal and human studies have suggested that Ca, Fe, Cu, Mg, and Zn are associated with hypochromia, febrile seizure, rickets, inﬂammatory processes, cardiovascular diseases, anemia, and other essential elements of metabolism (1, 2). Therefore, the assessment of trace element levels is helpful for understanding the body's nutritional status and assisting in the diagnosis of clinical diseases.

Every laboratory test needs a reference limit to properly assess the diagnosis. Approximately 80% of clinical decisions rely on reference intervals (RIs), which usually indicate the central 95% distribution within a healthy population (4). Generally, the lower reference limit and the upper reference limit correspond to the 2.5th to 97.5th percentiles of the distribution of test results (although there are exceptions in specific cases) (5). The International Federation of Clinical Chemistry (IFCC) and the Clinical Laboratory Standards Institute (CLSI) of the United States point out that the method of establishing RIs by establishing exclusion criteria and selecting appropriate reference individuals is the standard method for establishing RIs (6). However, its establishment process is cumbersome, expensive, and difficult to promote. Therefore, most hospitals refer to the RIs recommended by the kit instructions or textbooks (7). The indirect method uses the existing data in the laboratory information system and uses mathematical-statistical models to establish RIs. It can obtain similar results to the direct method, avoid the cumbersome process, and greatly optimize the cost-effectiveness. The defect of the indirect method to establish RIs is the inclusion of abnormal data of pathological conditions; however, through the collection and extraction of big data, normality transformation, appropriate outlier elimination rules, and mathematical-statistical models, most outliers can be identified and eliminated. The RIs obtained by this method have a high degree of credibility. Previously, using the data of the physical examination population, the RIs obtained by the BOX-COX transformation-interquartile range method-outlier elimination-Hoffmann linear fitting approach was almost consistent with the national standard results (8). This shows that the indirect method is feasible and reliable to establish RIs. At present, the majority of clinical laboratories in China utilize RIs derived from manufacturer package inserts, textbooks, or literature (9). Nevertheless, the robustness of these reference limits is open to question. A number of population features, including age, sex, and ethnicity, contribute to changes in analyte concentrations. It is, therefore, important to consider them when defining reference limits (4). Pediatric RIs specifically reflect the physiological states of children and adolescents throughout their growth and development. As a result, age and sex stratification are critical consideration in establishing pediatric RIs.

It is a challenge for clinical laboratories to establish RIs for infants and children based on the local population. The determination of pediatric RIs requires a significant investment of resources, as well as at least 120 healthy individuals in each subgroup (10). Despite the establishment of pediatric RIs based on healthy children and adolescents by several national and international initiatives, there are still discrepancies in population, sampling technique, and analytical procedure. It is, therefore, imperative to establish pediatric RIs that are suitable for the Chinese population. We established age-based and sex-based RIs using data collected at three health screening centers.

## 2 Materials and methods

### 2.1 Study population

The hospital population can be considered as a main population consisting of patients with normal laboratory findings and a small disordered population with pathology. They can be separated mathematically if the main population distribution is known (parametric approach) and the sample size is large enough, either by exclusion criteria or both. A total of 4,868 individuals from the three health screening centers were included, with 540, 2,728, and 1,600 participants from each center, respectively. Patients with clearly diagnosed micronutrient deficiencies or increases and participants lacking micronutrient reports were excluded. The project was approved by the Ethics Committee. Ethic approval code: 2024002.

### 2.2 Method and instruments

We examined the levels of trace elements by analyzing whole blood samples. Throughout the collection and processing of samples, we took every precaution to prevent contamination. Atomic absorption spectrometry was employed to measure the blood concentrations of Cu, Zn, Ca, Mg, Fe, and Pb. The BOHUI 5100 analyzer was utilized for detecting concentrations of Cu, Zn, Ca, Fe, and Mg, whereas the BOHUI 2100 analyzer was used specifically for Pb detection. Relevant reagents and calibration standards were sourced from Bohui Innovation Technology Co., Ltd (Beijing, China). Blood Pb concentrations were traceable to the national reference materials for Pb (GBW08619). Blood Cu, Zn, Ca, Mg, and Fe concentrations were traceable to the following national reference materials: GBW08615 (Cu), GBW08620 (Zn), GBW(E)080118 (Ca), GBW(E)080126 (Mg), and GBW08616 (Fe). Intra-assay and inter-assay coefficients of variation for trace element determination were presented in Table 1.

**Table 1:** Intra-assay and inter-assay coefficients of variation for trace element determination.

Variables	Intra-assay coefficients of variation	Inter-assay coefficients of variation
Cu	1.27%–6.50%	3.27%–8.51%
Zn	1.42%–11.49%	3.39%–13.50%
Ca	1.54%–2.03%	2.65%–3.77%
Mg	1.32%–3.02%	1.69%–3.87%
Fe	0.38%–1.42%	0.91%–2.69%
Pb	7.43%–11.46%	8.29%–12.55%

### 2.3 Statistical analysis

Continuous variables are presented as medians (interquartile range, IQR), and categorical variables are reported as numbers and percentages. Tukey's method was used to examine and remove outliers, and this process was repeated until no outliers were identified (11). We divided the participants into subgroups according to age and sex, with an interval of 1 year. We used the CART method to construct a decision tree to determine the optimal cutoff point for age the fundamental concept behind CART involves recursively partitioning the input space into smaller regions based on the values of various features or attributes of the data. Throughout this partitioning process, CART seeks optimized cutting criteria to form tree-shaped decision rules that minimize prediction errors (12, 13). The maximum depth of the decision tree was set to 2, and two methods were used to assess the appropriateness of the age cutoff. Differences between continuous subgroups were compared with the *Z* test according to the Harris–Boyd method. The value of z was formulated by the equation: $Z=\frac{X1-X2}{\sqrt{\left(\frac{s1^{2}}{n1}\right)+\left(\frac{s2^{2}}{n2}\right)}}$. The critical value compared to z was calculated by the equation of $Z^{*}\;=\;3\sqrt{\frac{n1+n2}{240}}$. if the Z value exceeds Z*, division is recommended. In addition, if the larger SD in one subgroup is more than 1.5 times the smaller SD in the next subgroup, the Harris-Boyd approach recommends splitting subgroups. The other approach to statistical testing means differences between subgroups are based on Lahti's proportionality criterion (14). In this method, data from two neighboring subgroups are used to calculate the lower limits (2.5th percentile) and upper limits (97.5th percentile). The out-of-range value is then calculated as the proportions of the distribution of the sub-groups outside the reference limits, which are determined by the combination of the neighboring sub-groups. If at least one of the four proportions of the subgroups outside the common reference limits is greater than or equal to 4.1% or less than or equal to 0.9%, a split will be advised (14). If all three methods above show that node division is unreasonable, adjacent subsets are merged. Otherwise, we will use the decision tree to subdivide the subset further and repeat the above steps.

Once the appropriate segmentation points had been identified, the refineR algorithm was used to calculate the RIs. This algorithm uses an inverse approach to identify the model that best explains the non-pathological distribution. R packages are obtained from CRAN (https://CRAN.Rproject.org/pack-age=refineR). We calculated the lower, upper, and median reference values for each age subgroup and the subset divided by the best-dividing point, as well as their respective 95% confidence intervals (CI). Finally, the Q value is clarified based on the relationship between trace elements and age.

Data were analyzed using SPSS (26.0 SPSS, IBMCorp) and R4.3.1, and a two-tailed *P* < 0.05 was considered statistically significant.

## 3 Results

### 3.1 General characteristics

Of the 4,868 people included, 935 were excluded because of the definite diagnosis of the lack or increase of trace elements. In total, 3,933 samples were analyzed to investigate the distribution of trace elements in age groups from birth to age 18. The number of boys was 2,215 and the number of girls was 1,718. The median blood concentrations of Cu, Zn, Ca, Mg, Fe and Pb were 19.74 μmol/L, 74.80 μmol/L, 1.60 mmol/L, 1.52 mmol/L, 7.84 mmol/L and 126.09 mmol/L, respectively (Tables 2, 3).

**Table 2:** Distribution characteristics of trace elements by age subgroup.

Group	Total	Female	Male
0 to <1 year	189 (4.81%)	97 (51.32%)	92 (48.68%)
1 to <2 years	698 (17.75%)	307 (43.98%)	391 (56.02%)
2 to <3 years	430 (10.93%)	183 (42.56%)	247 (57.44%)
3 to <4 years	400 (10.17%)	169 (42.25%)	231 (57.75%)
4 to <5 years	382 (9.71%）	160 (41.88%)	222 (58.12%)
5 to <6 years	278 (7.07%)	125 (44.96%)	153 (55.04%)
6 to <7 years	269 (6.84%)	127 (47.21%)	142 (52.79%）
7 to <8 years	265 (6.74%)	112 (42.26%)	153 (57.74%)
8 to <9 years	226 (5.75%)	108 (47.79%)	118 (52.21%)
9 to <10 years	231 (5.87%)	88 (38.10%)	143 (61.90%)
10 to <11 years	188 (4.78%)	76 (40.43%)	112 (59.57%)
11 to <12 years	146 (3.71%)	55 (37.67%)	91 (62.33%)
12 to <13 years	132 (3.36%）	59 (44.70%）	73 (55.30%)
13 to <14 years	76 (1.93%)	35 (46.05%)	41 (53.95%)
14 to <15 years	16 (0.41%)	11 (68.75%)	5 (31.25%)
15 to <16 years	7 (0.18%)	6 (85.71%)	1 (14.29%)
Total	3,933(100%)	1,718(43.68%)	2,215(56.32%)

**Table 3:** Characteristics of participants included.

Variables	Median (25th, 75th)
Age, years	4.00 (2.00, 8.00)
Copper (Cu), μmol/L	19.74 (15.77, 23.54)
Zinc (Zn), μmol/L	74.80 (65.48, 83.42)
Calcium (Ca), mmol/L	1.60 (1.54, 1.71)
Magnesium (Mg), mmol/L	1.52 (1.42, 1.63)
Iron (Fe), mmol/L	7.84 (7.36, 8.35)
Lead (Pb), mmol/L	126.09 (64.73, 203.74)

### 3.2 Age- and sex-specific RIs

**Figure 1:**
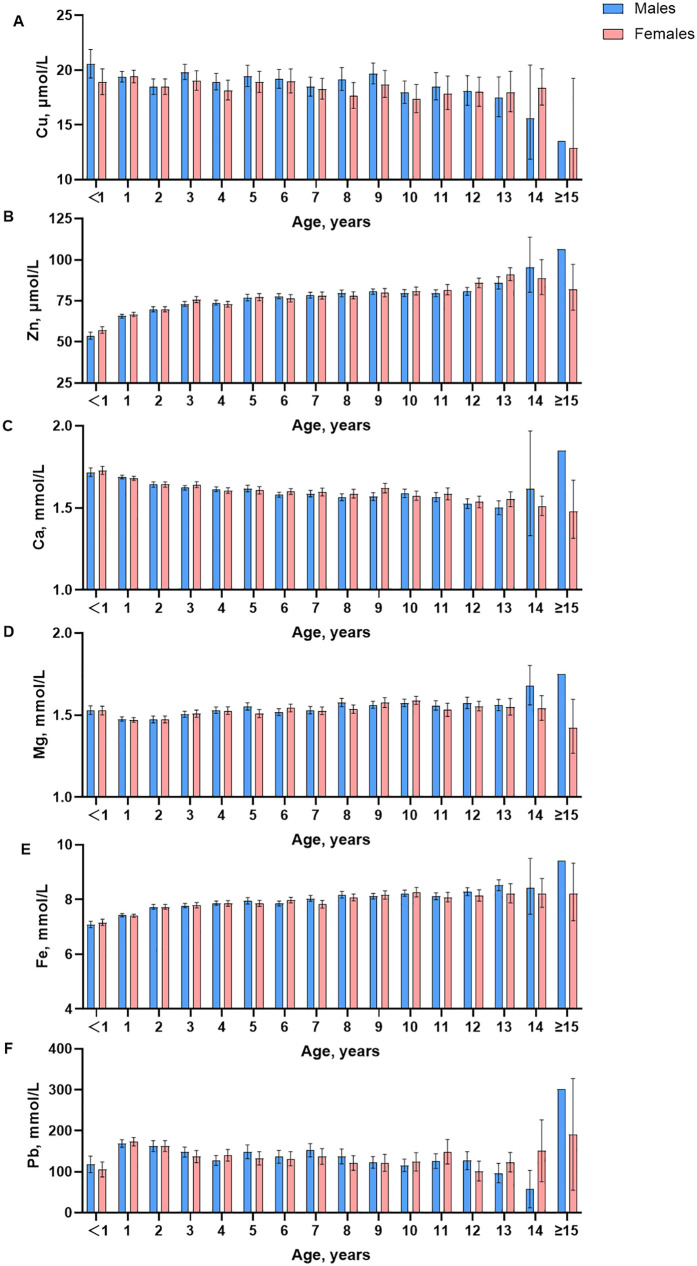
Box-plots of trace element for age and gender in different stages.

Box plots (Figure 1) show a visual examination of the distribution of trace elements. There are differences in trace element test results depending on age and gender. Decision trees were constructed by CART to determine the optimal age segmentation point (Supplementary Figure S1–S6). Supplementary Table S4 shows the statistical test using the Harris-Boyd method for age subgroups by sex. Z does not exceed z∗, and the division is reasonable. Furthermore, the Lahti method was also used to test the validity of age partitioning for the estimation of RIs. Supplementary Table S2 shows that at least one of the observed proportions in two subgroups is greater than 4.1% or less than 0.9%, indicating that the age partitioning is reasonable. Age- and sex-specific RIs were obtained by refineR calculation (Table 4).

**Table 4:** Medians and lower and upper reference limits for in age- and sex- specific groups divided by the adjusted optimal segmentation points.

Variables	Gender	Age	Total	Median (95%CI)	Lower limit (95%CI)	Upper limit (95%CI)
Cu	Males	3 mths–11 mths	92	16.84 (15.58, 24.56)	13.52 (8.68, 22.80)	20.93 (18.25, 30.91)
		1 yrs–8 yrs	1,657	20.29 (19.35, 20.93)	12.46 (8.59, 14.85)	30.62 (26.84, 31.43)
		9 yrs	143	21.23 (17.10, 24.14)	15.96 (9.36, 21.94)	25.98 (21.48, 30.76)
		10 yrs–	323	18.70 (14.37, 23.24)	13.18 (6.33, 20.10)	25.66 (18.32, 31.76)
	Females	2 mths–1 yrs	404	20.32 (17.66, 21.29)	15.99 (9.12, 17.79)	24.37 (21.96, 31.01)
		2 yrs–	1,314	19.87 (19.16, 20.01)	8.61 (7.06, 12.44)	30.39 (28.78, 31.19)
Zn	Males	3 mths–11 mths	92	53.17 (46.68, 61.30)	46.25 (29.83, 56.64)	60.57 (54.22, 71.29)
		1 yrs	391	66.89 (60.13, 77.64)	54.52 (47.84, 70.74)	79.94 (68.73, 87.24)
		2 yrs	247	68.01 (63.98, 77.99)	55.85 (53.70, 71.33)	82.56 (70.95, 87.76)
		3 yrs–4 yrs	453	74.06 (72.19, 77.08)	56.23 (54.73, 66.42)	90.71 (85.41, 94.95)
		5 yrs–	1,032	78.91 (75.85, 81.19)	61.12 (56.38, 63.38)	100.32 (91.38, 103.08)
	Females	2 mths–11 mths	97	54.07 (51.49, 60.29)	47.76 (43.71, 57.23)	61.12 (56.48, 67.46)
		1 yrs	307	73.29 (63.20, 75.12)	63.20 (44.51, 67.94)	82.87 (76.32, 88.40)
		2 yrs	183	72.77 (59.82, 79.02)	60.95 (45.62, 69.50)	86.28 (67.86, 96.95)
		3 yrs–11 yrs	1,020	76.73 (73.29, 83.41)	58.36 (52.98, 66.75)	100.21 (93.11, 111.89)
		12 yrs–	111	81.43 (78.16, 98.59)	78.14 (71.42, 91.60)	84.88 (81.67, 106.99)
Ca	Males	3 mths–1 yrs	483	1.68 (1.63, 1.73)	1.52 (1.50, 1.61)	1.86 (1.74, 1.89)
		2 yrs–5 yrs	853	1.58 (1.57, 1.58)	1.48 (1.47, 1.49)	1.67 (1.65, 1.70)
		6 yrs–	897	1.52 (1.50, 1.53)	1.41 (1.39, 1.44)	1.63 (1.56, 1.66)
	Females	2 mths–11 mths	97	1.70 (1.69, 1.76)	1.60 (1.55, 1.65)	1.81 (1.76, 1.90)
		1 yrs–	1,621	1.57 (1.56, 1.59)	1.44 (1.41, 1.47)	1.70 (1.66, 1.78)
Mg	Males	3 mths–	2,215	1.52 (1.50, 1.55)	1.27 (1.22, 1.33)	1.77 (1.75, 1.83)
	Females	3 mths–	1,718	1.52 (1.48, 1.54)	1.27 (1.17, 1.36)	1.77 (1.71, 1.79)
Fe	Males	3 mths–11 mths	92	6.52 (6.35, 7.42)	6.24 (6.12, 7.26)	6.79 (6.44, 7.58)
		1 yrs	391	7.70 (6.81, 7.85)	7.02 (6.47, 7.27)	8.36 (7.08, 8.77)
		2 yrs–6 yrs	995	7.80 (7.72, 7.95)	6.75 (6.59, 7.00)	9.01 (8.76, 9.16)
		7 yrs–	737	8.27 (7.99, 8.50)	7.24 (6.96, 7.57)	9.30 (9.05, 9.48)
	Females	2 mths–1 yrs	404	7.54 (6.77, 7.67)	6.81 (6.32, 7.15)	8.31 (7.16, 8.51)
		2 yrs–7 yrs	876	8.08 (7.53, 8.26)	7.05 (6.73, 7.45)	9.04 (8.31, 9.37)
		8 yrs–	438	8.22 (7.93, 8.36)	7.16 (6.76, 7.52)	9.30 (8.95, 9.54)
Pb	Males	3 mths–	2,215	27.72 (22.49, 33.07)	2.79 (1.44, 10.55)	85.75 (55.53, 106.84)
	Females	2 mths–11 mths	97	10.53 (8.89, 17.24)	5.91 (2.13, 12.87)	16.16 (12.09, 29.92)
		1 yrs–	1,621	25.39 (21.32, 34.18)	1.55 (1.23, 11.46)	68.62 (50.28, 82.74)

Cu, copper; Zn, zinc; Ca, calcium; Mg, magnesium; Fe, iron; Pb, lead; mths, months; yrs, years.

#### 3.2.1 Cu

Visual examination showed a significant decrease in content after the age of 15 years. An assessment by the Harris and Boyd method found the age partitions for Cu were 3 months to 11 months, 1 year, 2 years to 5 years, 6 years to 8 years, 9 years, 10 years and above in male children, and were 2 months to 11months, 1 year, 2 years, 3 years to 6 years, 7 years and above in female children. For trace element Cu, the RIs of boys at the age of 3–11 months, 1–8 years, 9 years, 10 years, and above are 13.52–20.9 μmol/L, 12.46–30.62 μmol/L, 15.96–25.98 μmol/L and 13.48–25.66 μmol/L, respectively. The RIs of girls aged 2–1 years, 2 years, and above are 15.99–24.37 μmol/L and 8.61–30.39 μmol/L.

#### 3.2.2 Zn

Visual examination showed the contents of Zn increased slightly with age, and in female infants and children, it was slightly lower than in men. The age partitions for Zn were 3 months to 1 year, 2 years, 3 years, 5 years, 5 years and above in male children and were 2 months to 11 months, 1 year, 2 years, 3 years, 4 years, 5 years to 8 years, 9 years to 11 years, 12 years, 13 years and above in female children. For trace element Zn, the RIs of boys at the age of 3–11 months, 1 year, 2 years, 3–4 years, 5 years, and above are 46.25–60.57 μmol/L, 54.52–79.94 μmol/L, 55.85–82.56 μmol/L, 55.23–90.71 μmol/L, and 61.12–100.32 μmol/L, respectively. The RIs of girls at the age of 2–11 months, 1 year, 2 years, 3–11 years, 12 years and above are 47.76–61.12 μmol/L, 63.20–82.87 μmol/L, 60.95–86.28 μmol/L, 58.36–100.21 μmol/L, and 78.14–84.88 μmol/L, respectively.

#### 3.2.3 Ca

Visual examination of the distribution for trace elements is shown in box plots (Figure 1). There are differences in trace element test results depending on age and gender. The contents of Ca decreased slightly, and in female infants and children, were slightly lower than those in men. The age partitions for Ca were 3 months to 1 year, 2 years, 3 years to 5 years, 6 years to 11 years, 12 years and above in male children and were 2 months to 11 months, 1 year, 2 years to 3 years, 4 years to 11 years, 12 years and above in female children. For trace element Ca, the RIs of boys at the age of 3 months −1 year, 2–5 years, 6 years and above are 1.52–1.86 mmol/L, 1.48–1.67 mmol/L, and 1.41–1.63 mmol/L, respectively. The RIs of girls at the age of 2 months-11 months, 1 year, and above are 1.60–1.81 mmol/L and 1.44–1.70 mmol/L.

#### 3.2.4 Mg

Visual examination showed the contents of Mg were relatively stable, and in female infants and children, they were slightly lower than those in men. The age partitions for Mg were 3 months to 3 years, 4 years to 7 years, 8 years and above in male children and were 2 months to 11 months, 1 year to 3 years, 4 years to 8 years, and 9 years and above in female children. For trace element Mg, the RIs of boys and girls at 3 months and above are 1.27–1.77 mmol/L.

#### 3.2.5 Fe

Visual examination showed the contents of Fe increased slightly with age, and in female infants and children, were slightly lower than those in men. The age partitions for Fe were 3 months to 11 months, 1 year, 2 years, 3 years, 4 years to 6 years, 7 years and above in male children and were 2 months to 11 months, 1 year, 2 years to 3 years, 4 years to 7 years, 8years, 9 years and above in female children. For trace element Fe, the RIs of boys at the age of 3 months-11 months, 1 year, 2–6 years, and 7 years and above are 6.24–6.79 mmol/L, 7.02–8.36 mmol/L, 6.75–9.01 mmol/L and 7.24–9.30 mmol/L, respectively. The RIs of girls at the age of 2 months—1 year, 2–7 years, 8 years, and above are 6.81–8.31 mmol/L, 7.05–9.04 mmol/L, and 7.16–9.30 mmol/L, respectively.

#### 3.2.6 Pb

Visual examination showed a significant decrease in content after the age of 15 years. The age partitions for Pb were 3 months to 11 months, 1 year to 3 years, 4 years to 8 years, 9 years and above in male children and were 2 months to 11 months, 1 year, 2 years, 3 years to 7 years, 8 years and above in female children. For trace element Pb, the RIs of boys aged 3 months and above are 13.48–414.25 mmol/L. The RIs of girls aged 2 months-11 months, 1 year, and above are 28.55–78.07 mmol/L and 7.49–331.50 mmol/L, respectively.

## 4 Discussion

Infants and children represent a fascinating demographic since they grow much faster than adults and require more significant amounts of nutrients, including the trace elements. Nevertheless, an overabundance of essential elements may cause health issues. This research determined the RIs for blood levels of Cu, Zn, Ca, Mg, Fe, and Pb, tailored by age and sex, based on a generally healthy cohort of infants and children. These RIs offer a more accurate interpretation for diagnosing and prognosticating pediatric clinical conditions.

The interpretation of clinical results relies significantly on the utilization of RIs (15). Nevertheless, the reporting of RIs for infants and children is infrequent. It is noteworthy that considerable variation was observed in this population, as blood concentrations of trace elements are susceptible to influence by environmental exposures and physiological parameters (16). Therefore, it is essential for clinical laboratories to establish age- and sex-specific RIs for trace elements in infants and children.

The most critical aspect of establishing RIs is the size of the study population. To determine the 90% confidence intervals for the central 95% RIs, a minimum of 120 participants was necessary (17). Nonetheless, acquiring a sample of healthy infants and children presents significant challenges. Walton's study proposed that hospital-based RIs were the most appropriate for interpretation, as they were measured under conditions that were analogous to those experienced by the majority of patients (18). In total, 3,933 healthy infants and children (2,215 boys and 1,718 girls) were selected from the three health examination centers in Lincang.

The discrepancies between the findings of various studies can be attributed to differences in the methodologies employed, the instruments utilized, the reagents used, and the timing of sample collections. The RIs for blood Cu, Zn, Ca, and Pb were found to be lower than those reported by other studies (19–21). Conversely, the RIs for blood Mg were found to be higher than those reported by other studies (19–21).The impact of age and gender on blood element concentrations was investigated. Noteworthy differences in zinc levels were observed between girls and boys beyond the age of 15, which aligns with the findings reported by Dongarra et al. (19). Additionally, we identified a trend of decreasing blood calcium levels with advancing age, resembling the results shared by Marwaha et al. (22). However, a separate study did not report any changes in this regard (20). In the present study, there was little difference in Ca levels between boys and girls before the age of 14 years, whereas other studies (22, 23) have found significantly higher Ca levels in boys than in girls. The study also found that blood lead levels gradually decreased with age between 1 and 13 years old. Nevertheless, an increase in blood Pb levels with age was documented in other studies (19). Another study conducted in China, which employed the same instrumentation, reported results similar to ours. However, a notable difference was observed in blood Pb levels, which may be attributed to their use of a substitution value of 10 for data below 10 µg/L (24). Our study's findings reinforce the critical importance of delineating groups based on age and gender in establishing RIs in infants and children. Partitioning by age and sex is a commonly encountered issue in the context of pediatric RIs and other clinical laboratory indexes (4, 25).

The data from the clinical laboratory, which exclusively enrolls outpatients, could be utilized for research on age and sex partitioning. In previous studies (26, 27), the cutoff age was typically estimated through visual examination, which may be subject to subjectivity. Indeed, the application of data mining techniques could facilitate the generation of more precise decision-making processes. For instance, a decision tree methodology has been employed for the assessment and forecasting of conditions (28, 29). In the present study, a decision tree method was carried out to investigate the cut-off age when the trace elements showed a tendency to be age-dependent. Compared with visual examination, decision tree analysis can present not only the cut-off age but also the mean difference between the two subgroups, which may contribute to adjusting the age division for RIs estimation. However, the age cut-off point suggested by the decision tree analysis was only a preliminary suggestion of possible partitioning, and the Harris-Boyd and Lahti methods were used to assess the appropriateness of the age partitioning.

We recognize that our study has at least two limitations. Firstly, we relied solely on atomic absorption spectrometry to analyze the blood elements. As a result, these RIs may not be as applicable to clinical laboratories employing alternative detection methods. Secondly, the adolescent RIs for certain elements were not provided due to the insufficient number of subjects in that age category (15 years and older).

## 5 Conclusions

We have presented age- and sex-specific RIs for blood concentrations of Cu, Zn, Ca, Mg, Fe, and Pb in infants and children, as recommended by the CLSI and the IFCC guidelines. The RIs achieved for Cu, Zn, Ca, Mg and Fe may provide a guideline for appropriately supplementing infants with trace and essential elements. The RIs for Pb may also have a role to play in monitoring and diagnosing overexposure to Pb in the environment.

## Data Availability

The original contributions presented in the study are included in the article/Supplementary Material, further inquiries can be directed to the corresponding author.
