# Supplementary material for: Age- and sex-specific reference intervals for trace elements in infants and children: a multi-center study in Lincang, China
Source: Front Pediatr. 2025 Sep 29;13:1547429. doi: 10.3389/fped.2025.1547429 (PMC12515901; doi:10.3389/fped.2025.1547429)
Supplement: Supplementary file 1 [file Table1.docx]

Table 2. Statistical tests for age partitioning of reference intervals estimation by Harris-Boyd’s method and Lahti’s method

| metal | Males (n=2215) | | | | | | Females (n=1718) | | | | | |
| --- | --- | --- | --- | --- | --- | --- | --- | --- | --- | --- | --- | --- |
|  | Age | Z^a^ | Z*^b^ | SD ratio^c^ | OOR^d^-lower  (%) | OOR^d^-higher  (%) | Age | Z^a^ | Z*^b^ | SD ratio^c^ | OOR^d^-lower  (%) | OOR^d^-higher  (%) |
| Cu | 3 mths-11 mths | 2.17 | 8.42 | 1.18 | 1.1 | 8.7 | 2 mths-1 yrs | 1.57 | 6.62 | 1.04 | 0.7 | 3.2 |
|  | 1 yrs-9 yrs |  |  |  | 2.6 | 2.2 | 2 yrs-6 yrs |  |  |  | 3.4 | 2.2 |
|  | 1 yrs-9 yrs | 3.46 | 8.92 | 1.07 | 2.1 | 2.7 | 2 yrs-6 yrs | 2.35 | 7.02 | 1.01 | 2.0 | 2.6 |
|  | 10 yrs- |  |  |  | 5.0 | 1.9 | 7 yrs- |  |  |  | 3.3 | 2.2 |
|  | 3 mths-11 mths | 2.23 | 8.10 | 1.19 | 1.1 | 8.7 | 2 mths-11 mths | 0.48 | 3.89 | 1.14 | 2.1 | 3.1 |
|  | 1 yrs-8 yrs |  |  |  | 2.5 | 2.1 | 1 yrs |  |  |  | 2.6 | 2.3 |
|  | 1 yrs-8 yrs | 0.98 | 8.22 | 1.06 | 2.7 | 2.5 | 1 yrs | 2.23 | 4.29 | 1.09 | 2.0 | 3.3 |
|  | 9 yrs |  |  |  | 0.7 | 2.1 | 2 yrs |  |  |  | 3.3 | 1.1 |
|  | 9 yrs | 2.86 | 4.18 | 1.01 | 0 | 2.8 | 2 yrs | 1.31 | 5.35 | 1.22 | 2.2 | 0.5 |
|  | 10 yrs- |  |  |  | 3.7 | 2.2 | 3 yrs-6 yrs |  |  |  | 2.6 | 3.1 |
|  | 3 mths-11 mths | 2.04 | 7.08 | 1.20 | 1.1 | 6.5 |  |  |  |  |  |  |
|  | 1 yrs-5 yrs |  |  |  | 2.6 | 2.2 |  |  |  |  |  |  |
|  | 1 yrs-5 yrs | 1.51 | 7.88 | 1.02 | 2.7 | 2.7 |  |  |  |  |  |  |
|  | 6 yrs-8 yrs |  |  |  | 1.9 | 1.7 |  |  |  |  |  |  |
|  | 6 yrs-8 yrs | 1.53 | 4.57 | 1.04 | 2.4 | 2.4 |  |  |  |  |  |  |
|  | 9 yrs |  |  |  | 2.1 | 2.8 |  |  |  |  |  |  |
|  | 6 yrs-9 yrs | 2.57 | 5.74 | 1.04 | 1.6 | 2.5 |  |  |  |  |  |  |
|  | 10 yrs- |  |  |  | 4.3 | 2.8 |  |  |  |  |  |  |
|  | 3 mths-11 mths | 2.21 | 4.26 | 1.33 | 2.2 | 6.5 |  |  |  |  |  |  |
|  | 1 yrs |  |  |  | 2.6 | 1.5 |  |  |  |  |  |  |
|  | 1 yrs | 0.81 | 6.83 | 1.16 | 2.0 | 2.0 |  |  |  |  |  |  |
|  | 2 yrs-5 yrs |  |  |  | 2.7 | 2.7 |  |  |  |  |  |  |
| Zn | 3 mths-11 mths | 10.63 | 5.23 | 1.01 | 15.2 | 1.1 | 2 mths-11 mths | 9.50 | 4.69 | 1.11 | 7.2 | 1.0 |
|  | 1 yrs-2 yrs |  |  |  | 0.6 | 2.8 | 1 yrs-2 yrs |  |  |  | 1.6 | 2.7 |
|  | 1 yrs-2 yrs | 9.45 | 6.40 | 1.02 | 3.8 | 0.8 | 1 yrs-2 yrs | 14.91 | 7.52 | 1.07 | 6.3 | 0.2 |
|  | 3 yrs-4 yrs |  |  |  | 0.7 | 4.9 | 3 yrs-11 yrs |  |  |  | 0.6 | 3.5 |
|  | 3 yrs-4 yrs | 9.42 | 7.46 | 1.02 | 4.2 | 0 | 3 yrs-11 yrs | 8.75 | 6.51 | 1.00 | 2.6 | 1.2 |
|  | 5 yrs- |  |  |  | 1.8 | 3.6 | 12 yrs- |  |  |  | 0.9 | 14.4 |
|  | 3 mths-11 mths | 9.19 | 4.26 | 1.04 | 12 | 1.1 | 2 mths-11 mths | 8.09 | 3.89 | 1.09 | 7.2 | 1.0 |
|  | 1 yrs |  |  |  | 0.3 | 2.8 | 1 yrs |  |  |  | 1.0 | 2.9 |
|  | 1 yrs | 4.06 | 4.89 | 1.05 | 2.6 | 0 | 1 yrs | 2.91 | 4.29 | 1.02 | 3.6 | 1.0 |
|  | 2 yrs |  |  |  | 2.0 | 6.1 | 2 yrs |  |  |  | 0.5 | 4.9 |
|  | 2 yrs | 3.69 | 4.23 | 1.04 | 3.6 | 1.2 | 2 yrs | 4.44 | 4.38 | 1.04 | 3.3 | 1.6 |
|  | 3 yrs |  |  |  | 0.9 | 3.5 | 3 yrs-4 yrs |  |  |  | 1.8 | 2.4 |
|  | 3 yrs | 0.75 | 4.12 | 1.00 | 2.6 | 2.2 | 3 yrs-4 yrs | 5.31 | 6.18 | 1.02 | 5.2 | 0.6 |
|  | 4 yrs |  |  |  | 2.3 | 3.2 | 5 yrs-11 yrs |  |  |  | 1.6 | 3.3 |
|  |  |  |  |  |  |  | 5 yrs-11 yrs | 4.80 | 5.30 | 1.08 | 2.5 | 2.0 |
|  |  |  |  |  |  |  | 12 yrs |  |  |  | 1.7 | 6.8 |
|  |  |  |  |  |  |  | 12 yrs | 1.77 | 2.04 | 1.15 | 1.7 | 1.7 |
|  |  |  |  |  |  |  | 13 yrs- |  |  |  | 2.0 | 3.8 |
|  |  |  |  |  |  |  | 3 yrs | 2.34 | 3.51 | 1.05 | 2.4 | 3.0 |
|  |  |  |  |  |  |  | 4 yrs |  |  |  | 2.5 | 1.9 |
|  |  |  |  |  |  |  | 5 yrs-8 yrs | 3.34 | 5.09 | 1.08 | 2.3 | 1.7 |
|  |  |  |  |  |  |  | 9 yrs-11 yrs |  |  |  | 2.7 | 3.7 |
| Ca | 3 mths-1 yrs | 5.83 | 5.23 | 1.02 | 6.6 | 2.3 | 2 mths-1 yrs | 5.90 | 5.32 | 1.02 | 1.5 | 2.0 |
|  | 2 yrs |  |  |  | 3.2 | 2.0 | 2 yrs-3 yrs |  |  |  | 2.8 | 0.3 |
|  | 2 yrs | 2.54 | 5.66 | 1.01 | 2.4 | 4.0 | 2 yrs-3 yrs | 5.76 | 6.72 | 1.10 | 1.1 | 2.6 |
|  | 3 yrs-5 yrs |  |  |  | 1.7 | 1.7 | 4 yrs-11 yrs |  |  |  | 2.9 | 2.0 |
|  | 3 yrs-5 yrs | 7.37 | 7.46 | 1.14 | 0.8 | 1.7 | 4 yrs-11 yrs | 4.52 | 6.01 | 1.09 | 1.8 | 2.4 |
|  | 6 yrs- |  |  |  | 2.7 | 3.0 | 12 yrs- |  |  |  | 4.5 | 1.8 |
|  | 6 yrs-11 yrs | 3.67 | 5.74 | 1.13 | 1.7 | 1.7 | 2 mths-11 mths | 3.57 | 3.89 | 1.05 | 4.1 | 6.2 |
|  | 12 yrs- |  |  |  | 3.3 | 1.7 | 1 yrs |  |  |  | 1.6 | 0.7 |
|  |  |  |  |  |  |  | 1 yrs | 4.26 | 4.97 | 1.01 | 1.6 | 1.6 |
|  |  |  |  |  |  |  | 2 yrs-3 yrs |  |  |  | 3.1 | 2.6 |
| Mg | 3 mths-3 yrs | 6.04 | 7.82 | 1.00 | 3.1 | 1.6 | 2 mths-11 mths | 3.16 | 5.32 | 1.05 | 2.1 | 2.1 |
|  | 4 yrs-7 yrs |  |  |  | 1.0 | 2.4 | 1 yrs-3 yrs |  |  |  | 1.8 | 2.3 |
|  | 4 yrs-7 yrs | 4.74 | 6.86 | 1.04 | 2.7 | 1.9 | 1 yrs-3 yrs | 6.07 | 6.96 | 1.03 | 3.0 | 1.4 |
|  | 8 yrs- |  |  |  | 1.4 | 2.4 | 4 yrs-8 yrs |  |  |  | 1.6 | 3.2 |
|  |  |  |  |  |  |  | 4 yrs-8 yrs | 3.59 | 6.01 | 1.02 | 2.7 | 2.5 |
|  |  |  |  |  |  |  | 9 yrs- |  |  |  | 1.2 | 2.1 |
| Fe | 3 mths-1 yrs | 6.55 | 5.23 | 1.10 | 2.9 | 0.4 | 2 mths-11 mths | 3.64 | 3.89 | 1.02 | 6.2 | 0 |
|  | 2 yrs |  |  |  | 1.2 | 6.5 | 1 yrs |  |  |  | 1.3 | 2.9 |
|  | 2 yrs | 4.01 | 6.11 | 1.03 | 2.8 | 0.4 | 1 yrs | 11.07 | 6.66 | 1.13 | 4.6 | 0 |
|  | 3 yrs-6 yrs |  |  |  | 2.3 | 2.9 | 2 yrs-7 yrs |  |  |  | 1.6 | 3.1 |
|  | 3 yrs-6 yrs | 9.60 | 7.46 | 1.02 | 3.3 | 0 | 2 yrs-7 yrs | 8.09 | 7.02 | 1.14 | 2.2 | 0.8 |
|  | 7 yrs- |  |  |  | 1.4 | 4.5 | 8 yrs- |  |  |  | 2.7 | 5.5 |
|  | 3 mths-11 mths | 4.95 | 4.26 | 1.02 | 4.3 | 1.1 | 2 yrs-3 yrs | 2.86 | 5.73 | 1.06 | 2.0 | 2.0 |
|  | 1 yrs |  |  |  | 2.0 | 2.3 | 4 yrs-7 yrs |  |  |  | 2.3 | 2.7 |
|  | 3 yrs | 2.32 | 5.30 | 1.05 | 2.6 | 0.4 | 8 yrs | 1.45 | 4.05 | 1.16 | 1.9 | 0 |
|  | 4 yrs-6 yrs |  |  |  | 2.3 | 3.3 | 9 yrs- |  |  |  | 2.4 | 2.4 |
| Pb | 3 mths-11 mths | 4.03 | 6.00 | 1.04 | 5.4 | 0 | 2 mths-11 mths | 6.31 | 4.69 | 1.00 | 7.2 | 2.1 |
|  | 1 yrs-3 yrs |  |  |  | 2.1 | 2.8 | 1 yrs-2 yrs |  |  |  | 1.4 | 2.4 |
|  | 1 yrs-3 yrs | 4.49 | 7.88 | 1.06 | 1.8 | 2.4 | 1 yrs-2 yrs | 6.06 | 6.66 | 1.05 | 1.4 | 2.7 |
|  | 4 yrs-8 yrs |  |  |  | 3.2 | 2.5 | 3 yrs-7 yrs |  |  |  | 2.9 | 2.3 |
|  | 4 yrs-8 yrs | 3.77 | 6.86 | 1.15 | 6.9 | 3.0 | 3 yrs-7 yrs | 1.95 | 6.51 | 1.01 | 1.9 | 2.7 |
|  | 9 yrs- |  |  |  | 2.4 | 1.5 | 8 yrs- |  |  |  | 3.0 | 2.1 |
|  | 3 mths-3 yrs | 6.05 | 9.11 | 1.00 | 1.9 | 2.5 | 1 yrs | 1.26 | 4.29 | 1.01 | 1.0 | 2.6 |
|  | 4 yrs- |  |  |  | 3.0 | 2.5 | 2 yrs |  |  |  | 4.9 | 2.2 |

Abbreviation: Cu, copper; Zn, zinc; Ca, calcium; Mg, magnesium; Fe, iron; Pb, lead; mths, months; yrs, years; OOR, out of range.

^a^ The z values are calculated by z =($\bar{x_{1}}$-$\bar{x_{2}}$)/[(s_1_^2^/n_1_)+(s_2_^2^/n_2_)]^1/2^.

^b^ The z* values are calculated by z* = 3[(n1 + n2) / 240]1/2.

^c^ SD ratios are computed by the larger SD divided by the smaller SD.

^d^ OOR are calculated as proportions of subgroups distribution outside reference limits established by combined neighboring subgroups.
