# Supplementary figures and images for: Age- and sex-specific reference intervals for trace elements in infants and children: a multi-center study in Lincang, China
Source: Front Pediatr. 2025 Sep 29;13:1547429. doi: 10.3389/fped.2025.1547429 (PMC12515901; doi:10.3389/fped.2025.1547429)

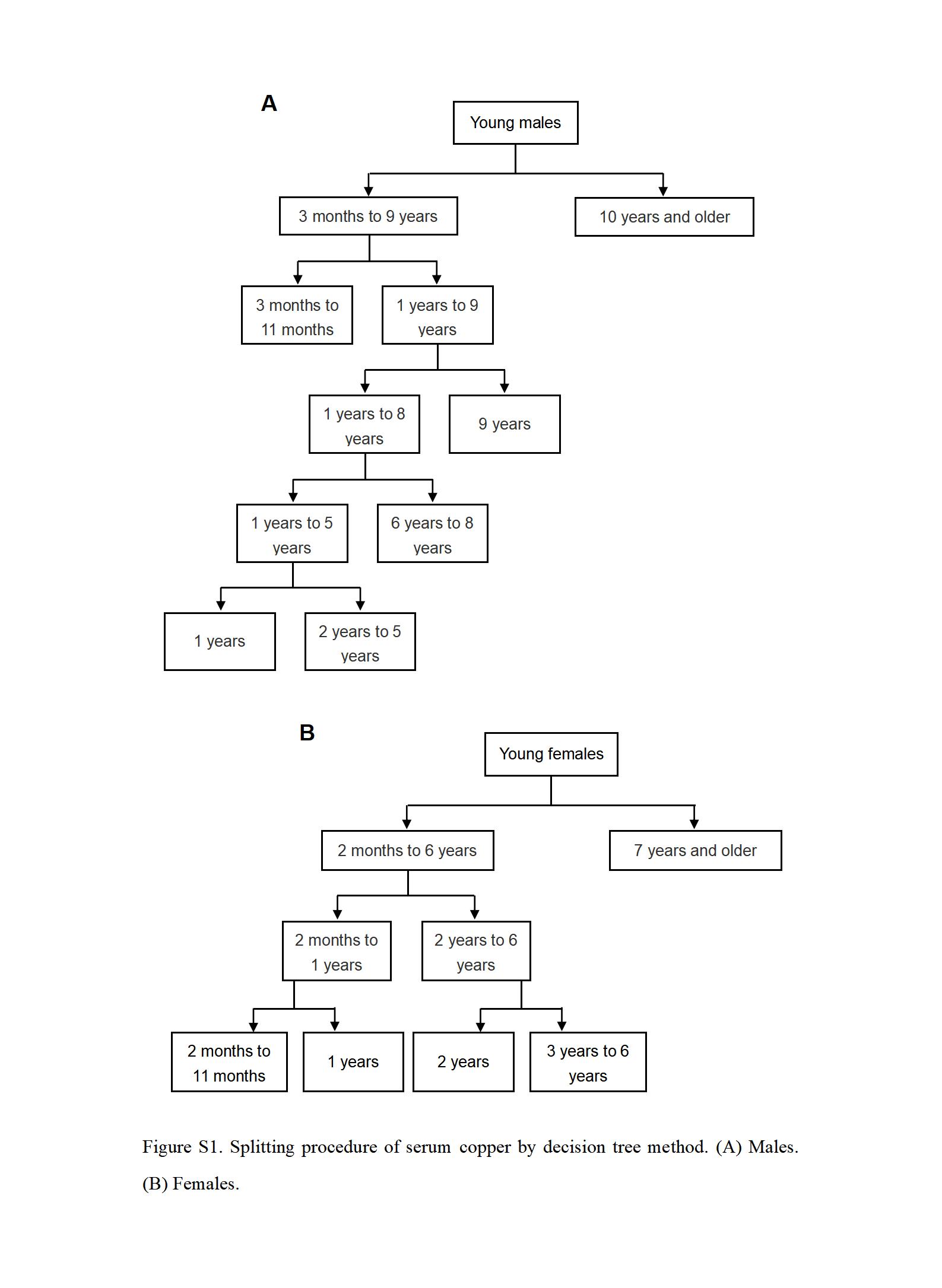

Supplement: Supplementary file 2 [file Image1.jpg]

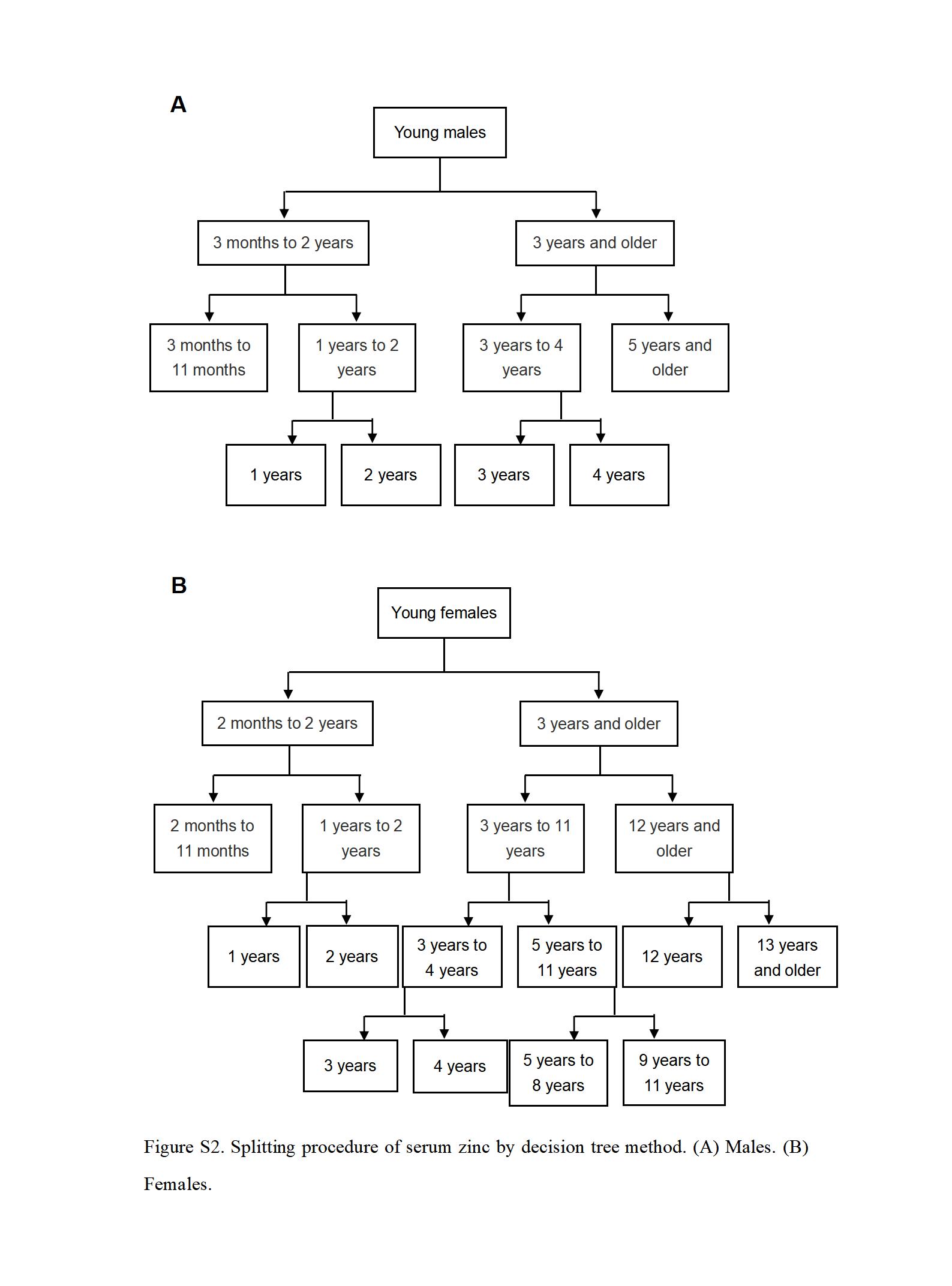

Supplement: Supplementary file 3 [file Image2.jpg]

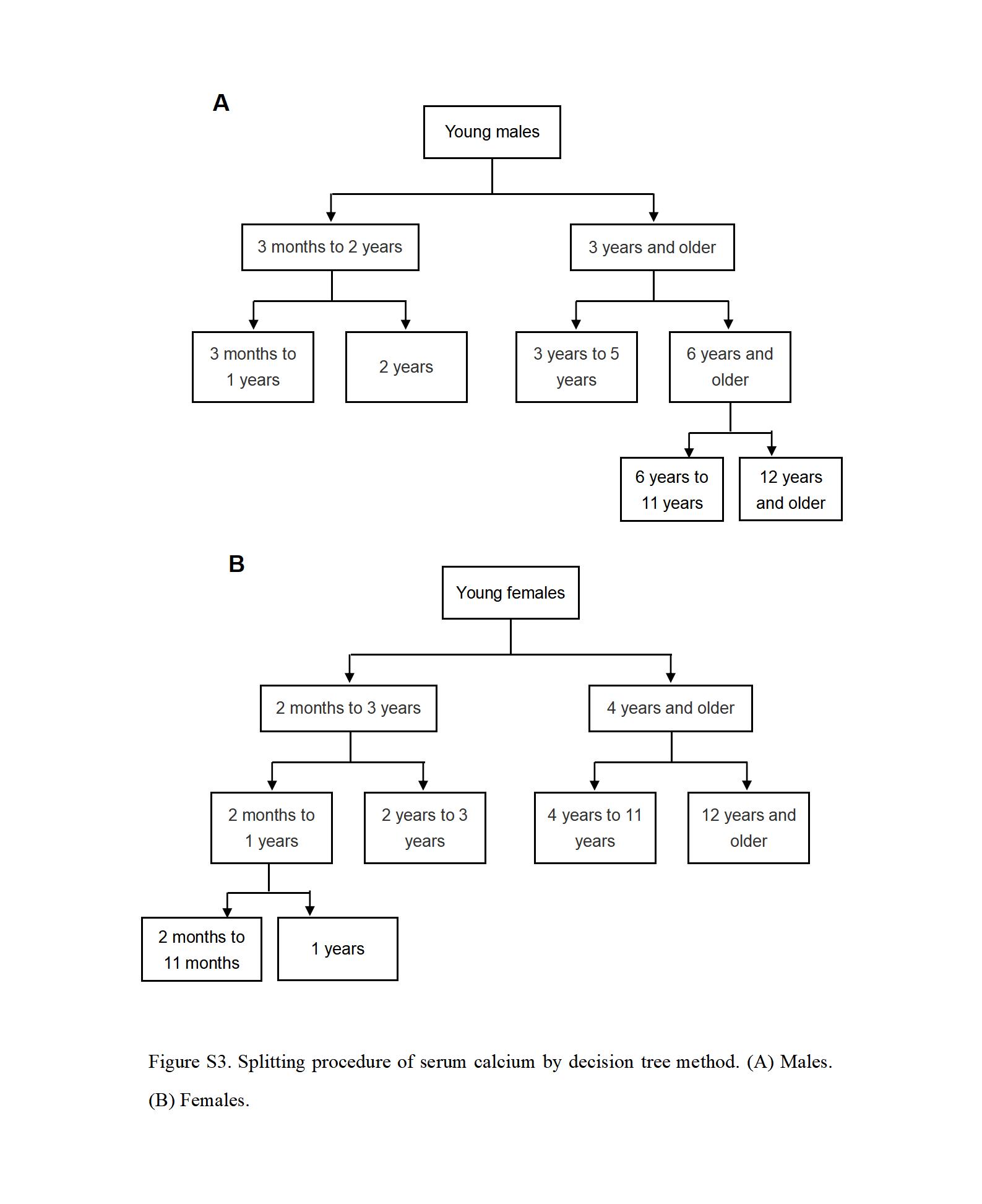

Supplement: Supplementary file 4 [file Image3.jpg]

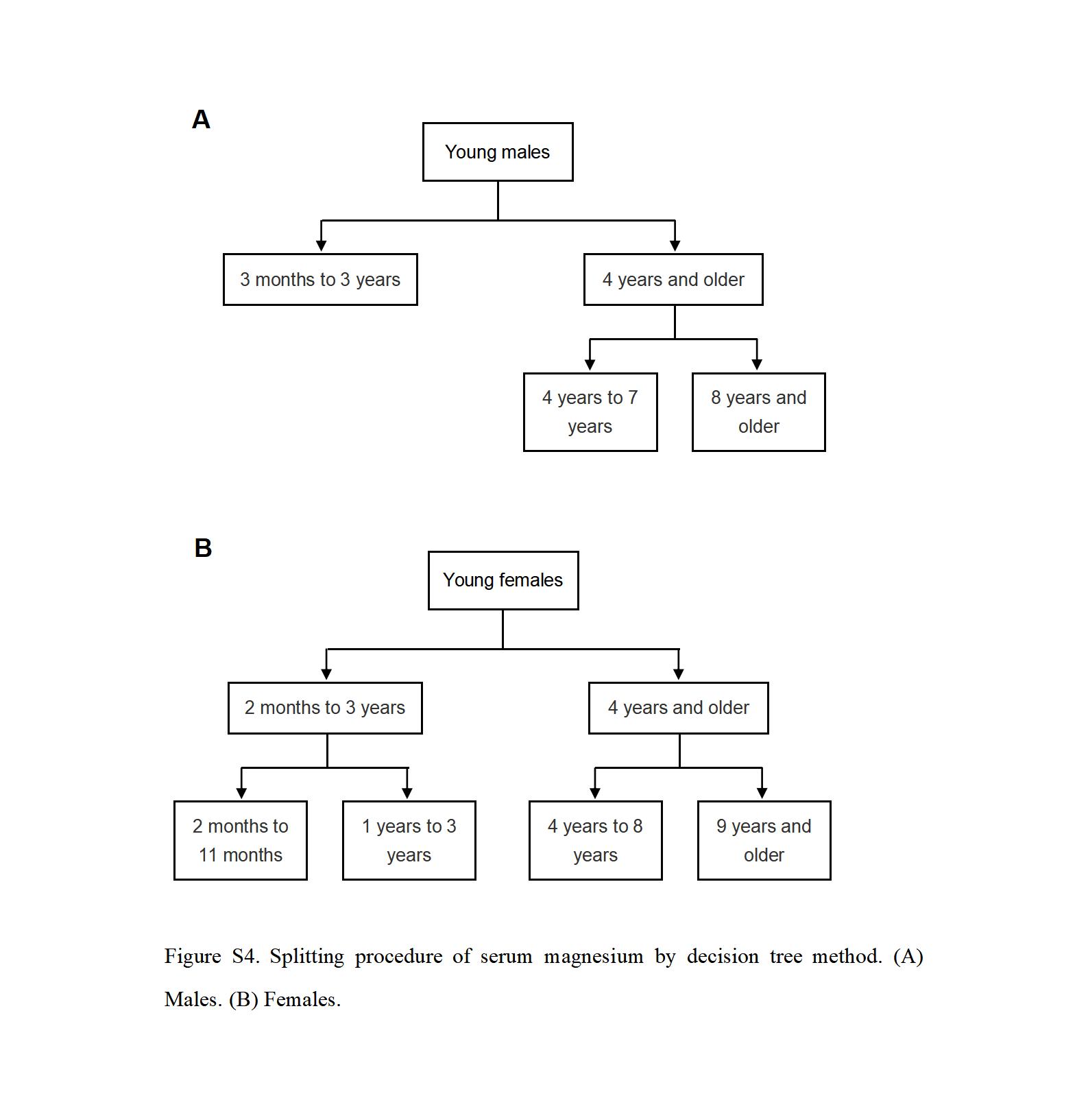

Supplement: Supplementary file 5 [file Image4.jpg]

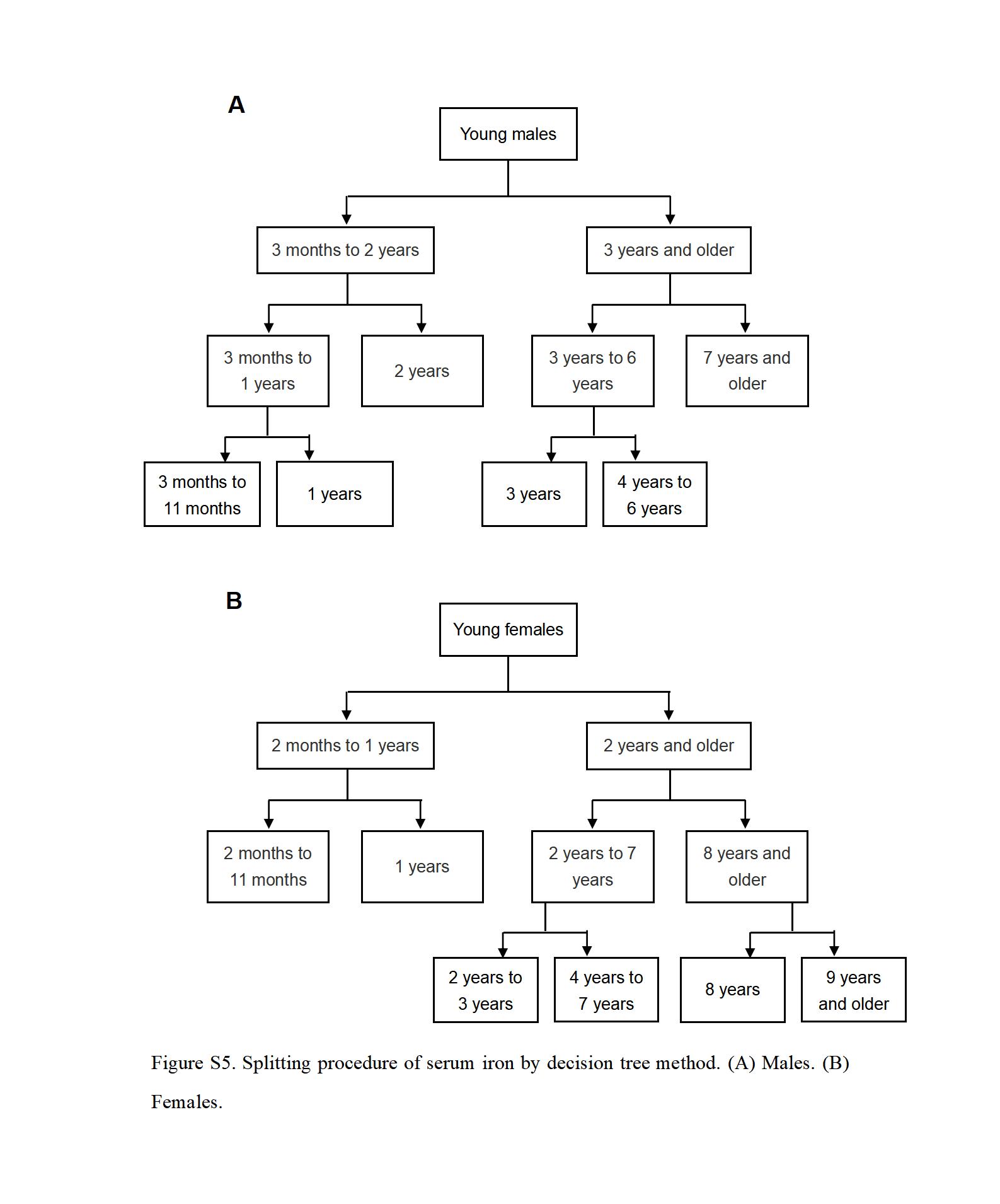

Supplement: Supplementary file 6 [file Image5.jpg]

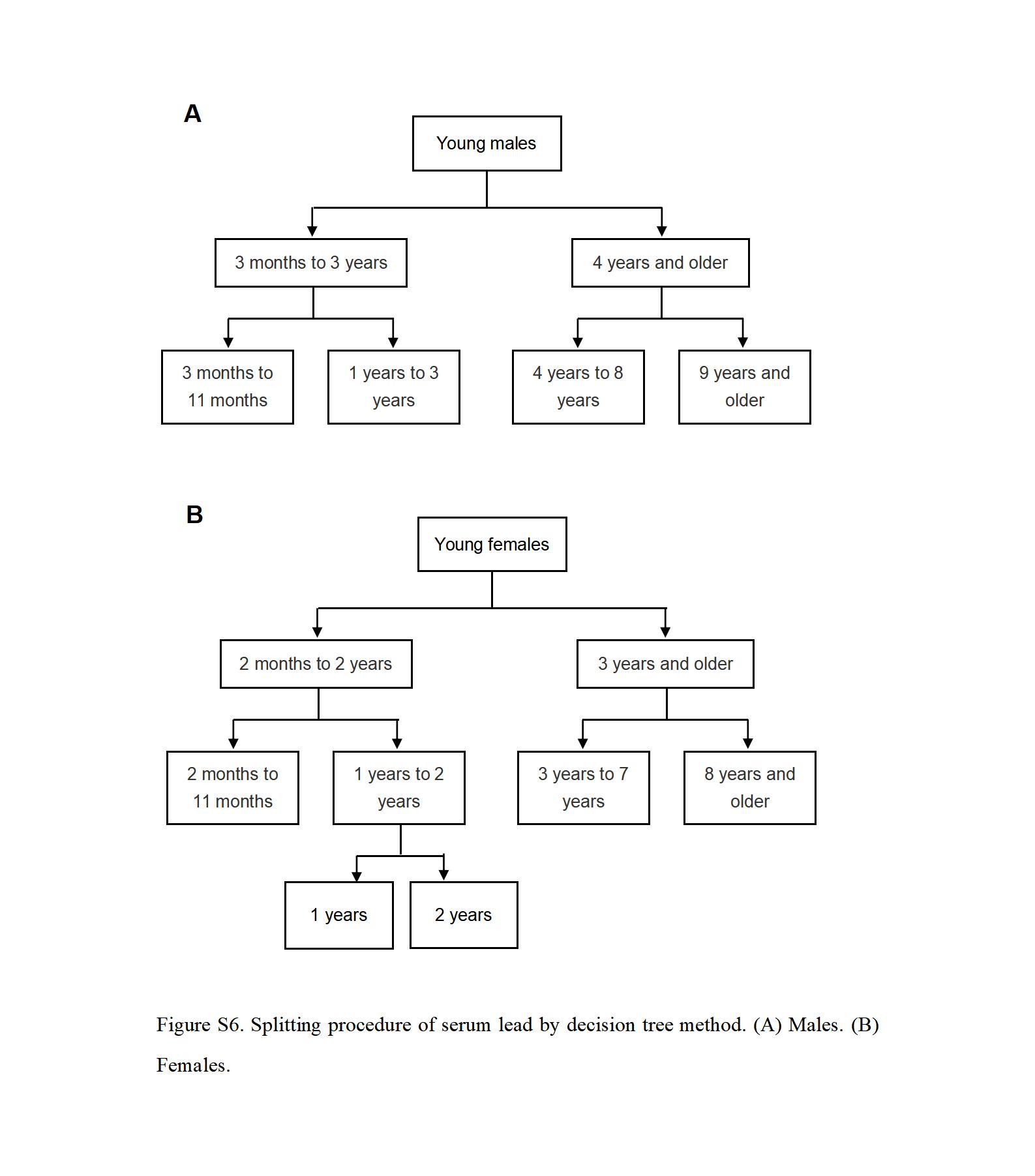

Supplement: Supplementary file 7 [file Image6.jpg]
